# Multidimensional Measures of Physical Activity and Their Association with Gross Motor Capacity in Children and Adolescents with Cerebral Palsy

**DOI:** 10.3390/s20205861

**Published:** 2020-10-16

**Authors:** Corinna N. Gerber, Lena Carcreff, Anisoara Paraschiv-Ionescu, Stéphane Armand, Christopher J. Newman

**Affiliations:** 1 Pediatric Neurology and Neurorehabilitation Unit, Department of Pediatrics, Lausanne University Hospital, 1011 Lausanne, Switzerland; lena.carcreff@hcuge.ch (L.C.); christopher.newman@chuv.ch (C.J.N.); 2 Laboratory of Kinesiology Willy Taillard, Geneva University Hospital and University of Geneva, 1205 Geneva, Switzerland; stephane.armand@hcuge.ch; 3 Laboratory of Movement Analysis and Measurement, Ecole Polytechnique Fédérale de Lausanne, 1015 Lausanne, Switzerland; anisoara.ionescu@epfl.ch

**Keywords:** cerebral palsy, inertial measurement units, physical activity pattern, capacity, performance

## Abstract

The current lack of adapted performance metrics leads clinicians to focus on what children with cerebral palsy (CP) do in a clinical setting, despite the ongoing debate on whether capacity (what they do at best) adequately reflects performance (what they do in daily life). Our aim was to measure these children’s habitual physical activity (PA) and gross motor capacity and investigate their relationship. Using five synchronized inertial measurement units (IMU) and algorithms adapted to this population, we computed 22 PA states integrating the type (e.g., sitting, walking, etc.), duration, and intensity of PA. Their temporal sequence was visualized with a PA barcode from which information about pattern complexity and the time spent in each of the six simplified PA states (PAS; considering PA type and duration, but not intensity) was extracted and compared to capacity. Results of 25 children with CP showed no strong association between motor capacity and performance, but a certain level of motor capacity seems to be a prerequisite for the achievement of higher PAS. Our multidimensional performance measurement provides a new method of PA assessment in this population, with an easy-to-understand visual output (barcode) and objective data for clinical and scientific use.

## 1. Introduction

Cerebral palsy (CP) is the most common cause of physical disability in childhood, with a neonatal prevalence of about 2:1000 live births [1]. CP results from non-progressive perinatal damage to the brain and leads to motor disorders with isolated or combined neurologic features including spasticity, dystonia, dyskinesia, and/or ataxia [2]. Motor disorders in CP are very heterogeneous and even though the brain damage underlying CP is non-progressive, after development towards a peak, gross motor function may decline in some children during later childhood and youth, while it remains relatively stable in others [3]. To counteract potential deterioration in functioning and to avoid negative secondary conditions such as pain, fatigue, and overweightness, children with CP should participate regularly in varied activities [4,5].

Physical activity (PA) is highly important for the development, health, and well-being, while sedentary behavior is associated with negative impacts on function and general health [6,7]. Children with CP have lower levels of PA than typically developing children, with increasing sedentary behavior the higher the level of motor impairment [8,9]. Most therapies are cost-intensive and the therapy time per person is limited. Therefore, to achieve a sufficient level of activity, it should be incorporated in patients’ lifestyles and promoted in natural, everyday settings [4].

For therapeutic decision making, standard assessments in children with CP typically include measures of body structure and body function, as well as the child’s capacity as described by the WHO [10], all measured in the clinical setting, while measures of performance are rarely used. However, whether a direct link between capacity (what a child is able to do in a standardized setting), capability (what a child can do in his/her daily situation), and performance (what a child actually does in the habitual environment) exists in children with CP remains an unsolved question [11,12,13,14].

While there are several valid and reliable tests for motor capacity that are part of routine clinical assessments, measures of motor performance are mainly questionnaire-based [15], which makes them subject to biases [16]. However, in recent years, inertial measurement units (IMUs) have emerged to accurately measure walking performance and PA parameters in children with CP [17,18,19,20]. In a recent study, Carcreff et al. [21] compared different setups using IMUs to measure spatio-temporal gait parameters in children with CP as compared with an optoelectronic system. While for typically developing children and children with a low degree of disability (Gross Motor Function Classification System (GMFCS) level I), a setup with two sensors showed good results, for children with higher levels of disability (GMFCS levels II–III), a setup with one sensor at each shank and thigh proved more robust [21]. With an additional sensor on the trunk, sedentary behavior and PA can be reliably measured [22].

Few studies have compared objectively measured daily life activity with motor capacity in children with CP. These used a single sensor (thigh-fixed Activ-PAL [23,24] (PAL Technologies, Glasgow, UK), ankle-fixed StepWatch [25] (Modus Health, Edmunds, WA, USA), or hip-fixed ActiGraph 7164 [26] (ActiGraph, Pensacola, FL, USA)) to estimate lying, sitting, standing, and walking (e.g., walking hours per day, number of steps per day, cadence) as a measure of physical activity. However, the limits of single-sensor setups to measure PA in patients with higher levels of impairment has been pointed out previously [27]. Furthermore, PA is a multidimensional construct that typically includes the type (e.g., standing), duration (e.g., total duration of sedentary periods), frequency (e.g., number of walking periods), and intensity (e.g., cadence) of activity. Additional dimensions such as the context (e.g., school/home) and pattern (e.g., changes between different activities) of PA provide further insight into an individual’s physical behavior. Paraschiv-Ionescu et al. reported the value of using a multi-sensor-setup in order to barcode human PA by integrating these diverse aspects of PA [28], and of its subsequent complexity analyses [29]. Validity of such barcoding and complexity analyses has been shown for patients with chronic pain [28] and well-functioning older adults [29].

In a recent literature review looking at studies that objectively measured daily life PA levels, Verschuren et al. [7] found that individuals with higher levels of impairment (i.e., GMFCS levels IV and V) did not achieve higher states of PA (i.e., moderate to vigorous PA). While the GMFCS is a gross motor classification system, a cut-off point on a continuous measure of motor capacity could be more revealing of the prerequisites to achieve certain levels of habitual PA.

The aims of our study were to (1) objectively measure PA of children and adolescents with CP using multidimensional measures (i.e., PA states (PAS) based on the barcoding method by Paraschiv-Ionescu et al. [28] and PA pattern complexity [29]), (2) to investigate the relationship between PA and gross motor capacity, and (3) to use gross motor capacity to determine cut-off levels needed to achieve a certain level of motor performance.

A certain relationship between capacity and performance can be expected as, for example, patients with very limited motor capacity most probably also show lower motor performance. However, while capability may be directly limited by the level of capacity, daily behavior (performance) might differ considerably due to other parameters such as environmental factors (e.g., furniture, walking aids, and other adaptations facilitating transfers and moving around in the habitual environment) or personal factors (e.g., intrinsic motivation, weight, age). Based on this assumption that gross motor capacity might only be one aspect influencing daily PA behavior, and due to a lack of comparable studies using PA pattern complexity computation, we expected fair correlations of PA pattern complexity and gross motor capacity. Based on previous literature [23,25,26], we hypothesized that PAS would correlate moderately (correlation coefficients 0.50–0.75) with gross motor capacity. We expected a negative association for the lowest and positive association for the highest PA levels. Furthermore, we expected higher gross motor capacity cut-off levels for higher PAS.

## 2. Materials and Methods

### 2.1. Participants

We performed cross-sectional analyses including participants from two other studies, a cross-sectional study (study 1) and a clinical trial (study 2). Both studies had received ethical approval by the local ethical committee (study 1, CCER-15-176; study 2, CER-VD-2016-01831), and written informed consent was obtained from participants 14 years or older and from their legal guardians for younger patients. The clinical trial registration ID was ISRCTN11365830 (ISRCTN registry).

Convenience samples meeting the following criteria were included: (i) diagnosis of CP; (ii) GMFCS [30] level I-III; (iii) ages between 8 and 20 years for study 1, between 7 and 18 years for study 2. Exclusion criteria for all participants were: (i) mental age <7 years (estimated by the referring pediatrician based on academic achievement or by previous IQ test); (ii) severe visual disorder; (iii) attention deficit, other significant behavioral issues, or suspected non-compliance that could compromise adequate participation in the studies. Participants were recruited from the patients followed at the pediatric orthopedics unit of the Geneva University Hospitals (HUG) for study 1 and from the pediatric neurology and neurorehabilitation unit of Lausanne University Hospital (CHUV) for study 2.

### 2.2. Protocol

The Gross Motor Function Measure 66 (GMFM-66) [31] was used to measure patients’ capacity. The GMFM is the most common assessment and “gold standard” for measuring gross motor functioning in children with CP [32,33]. The latest version of the GMFM, the GMFM-66, consists of 66 items measuring gross motor function in five dimensions: (A) lying and rolling, (B) sitting, (C) crawling and kneeling, (D) standing, and (E) walking, running, and jumping. Each item is scored on a 4-point scale. The Gross Motor Ability Estimator (GMAE) was used to compute overall GMFM-66 scores ranging from 0–100, with higher values indicating less impairment. A GMFM-certified human movement scientist performed the GMFM testing and scoring.

To measure daily life performance, a setup with five IMUs (described below) was used. Participants were asked to wear two IMUs on each thigh (anteriorly midway between hip and knee) and shank (laterally above the malleolus) and one on the trunk (on the midline at breast level) throughout a normal school day (if possible 10 consecutive hours). At the beginning of the day, the IMUs were placed by the parents or caregivers, who received practical training as well as a user guide to support them at home for the IMUs’ handling and placement (see Supplementary Document 1). Wrong placement of the sensors as well as, for example, accidental exchange of two sensors (e.g., thigh sensor at the ankle and vice-versa) could be corrected by manual assignment of sensor location and/or axes before running the analyses. Hypoallergenic double-sided hydrogel stickies (PAL stickies, PAL Technologies Ltd., Glasgow, UK) were used for the fixation, IMUs of the legs were further protected from falling with tight pants and high socks, and the trunk sensor with Mefix tape (Mölnlycke Health Care SA, 2600 Berchem, Belgium), if necessary. During the measurement day, patients followed their habitual activities such as school, leisure time, etc., until undressing in the evening.

### 2.3. Equipment and Data Processing

The IMUs used in this study were Physilog4^®^ sensors (Gait Up, 1020 Renens, Switzerland) containing a triaxial accelerometer, triaxial gyroscope, and barometer. The five IMUs were synchronized and sampling frequency was set at 100 Hz. Data were post-processed and analyzed using Matlab R2017 software (Mathworks, Natick, MA, USA). To auto-calibrate the sensors’ axis, a principal component analysis (PCA) was used [34]. The oriented pitch (i.e., in the sagittal plane) angular velocity of the shanks were used to detect walking episodes [35]. A minimum of 4 consecutive steps was needed to be considered as a relevant walking bout.

**Figure 1:**
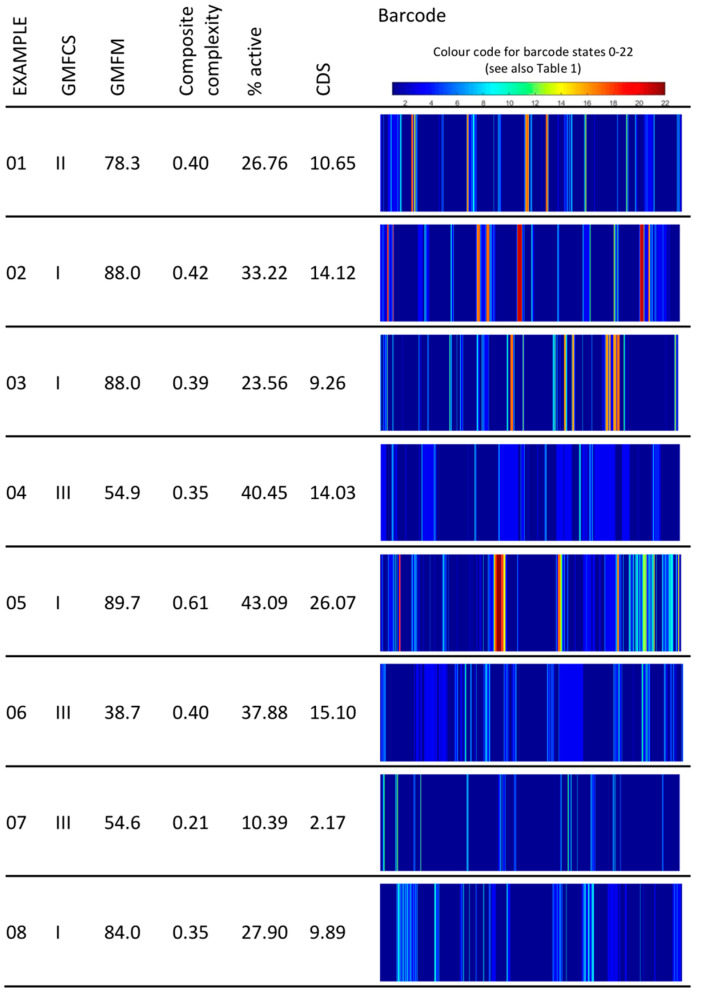
Example barcodes for children with different GMFCS levels and GMFM scores. The composite complexity is the sum of the three physical activity pattern complexity scores; information entropy (H), Lempel-Ziv complexity (LZC), and sample entropy (SampEn). % active is the portion of time participants spent in any activity from standing to highly intensive activity of very long duration (barcode states 3–22). Abbreviations: GMFCS, Gross Motor Function Classification Level; GMFM, Gross Motor Function Measure; CDS, composite deterministic score.

**Barcoding Physical Activity.** The trunk and thigh accelerations were used to detect lying/sitting and standing postures based on gravity acceleration (adapted from Paraschiv-Ionescu et al. [27]). Based on the refined approach for barcoding human PA in chronic pain condition by Paraschiv-Ionescu et al. [28], 22 barcode states were computed (see Table 1). The barcode states are defined combining different PA features, i.e., type, intensity, and duration of PA [28] and range from lying/sitting activities of low intensity (barcode state 1) to very long phases of walking/running and of very high intensity (barcode state 22). While for lying/sitting and standing body acceleration is used to determine intensity, cadence is used for active states of walking or running. Higher intensities of standing can, for example, be reached by playing table tennis or other activities with an upright position but less than 4 consecutive steps. To visualize the participants’ temporal sequence of barcode states throughout the day (PA pattern), the sequence was represented as a color barcode (see Figure 1).

**Physical Activity Pattern Complexity.** Paraschiv-Ionescu et al. [28] showed that the PA barcode contains important information about the variety, the temporal dynamic (i.e., ability to change between body postures/activities), and the duration of PA. Therefore, they proposed the calculation of PA complexity as a comprehensive metrics quantifying the amount and variety of barcode states and the temporal structure of the sequence [28]. The three resulting complexity metrics are: 1. the normalized information entropy (Hn) depicting the variety of barcode states present in the barcode (the more types of barcode states a person achieves, the higher the value of Hn, i.e., if only lying/sitting was achieved, Hn value results very low); 2. the Lempel-Ziv complexity *(LZC)* capturing the number of new sub-patterns discovered as the barcode progresses from the beginning to end; 3. the sample entropy (SampEn) computing the regularity of a time series by examining the presence of similar sub-patterns in the data sequence. Information entropy metric is more sensitive to variety of barcode states, whereas Lempel-Ziv and sample entropy capture both variety and temporal dynamic of barcode states [28]. Hn, LZC, and SampEn are all normalized (ranging from 0 to 1).

These complexity metrics, together with the %activity (all barcode states except for lying/sitting) can be combined to a composite complexity score, the composite deterministic score (CDS) [28], combining the overall PA pattern complexity to a composite complexity (CC) and including the overall duration of PA, as follows:

CC=Hn+SampEn+LZC

CDS=CC∗activity=(Hn+SampEn+LZC)∗activity

*CC* being the sum of Hn, SampEn, and LZC can range from 0 to 3. The value of CDS depends on %activity and can, theoretically, range from 0 * %activity to 3 * %activity. The higher the %activity and the higher the PA pattern complexity, the higher the value of CDS.

**Physical Activity States.** In order to get numerical values for clinical interpretation and to enable the determination of cut-off levels needed to achieve a certain level of motor performance, 6 PAS were derived from the 22 barcode states. The PAS are global metrics (i.e., % of the monitored time in a certain “activity state”) taking into consideration the type and duration (but not intensity) of PA and were defined as follows: 1. sitting/lying, 2. standing, 3. active short, 4. active moderate, 5. active long, and 6. active very long duration (see Table 1). The barcode states, PAS, respectively, were expressed as a percentage of the total measurement time.

For each PAS, participants were divided into achievers, i.e., participants that spent some of their time in the PAS, and non-achievers, i.e., participants that did not spend any time in the PAS.

To make results from this novel approach better comparable to previous literature, we used grouping variables for the active states; in one grouping variable, we summed up all active states (PAS 3–6), and in the other, we summed up only active states of at least moderate duration (PAS 4–6).

### 2.4. Statistical Analyses

Normality of distribution was tested with the Kolmogorov–Smirnov test.

To depict the relationship between gross motor capacity and physical daily life performance, we correlated GMFM-66 scores with PAS and pattern complexity using Pearson correlations in case of normal or Spearman’s rho in case of non-normal distribution of data. Correlations were interpreted as follows: 0.00–0.25 no to little; 0.25–0.50 fair; 0.50–0.75 moderate to good; 0.75–1.00 very good to excellent agreement [36].

To investigate the ability of the GMFM-66 score to discriminate between achievers and non-achievers for each PAS, we performed receiver operating characteristics (ROC) analysis. The highest Youden Index (= sensitivity + specificity −1) was extracted to depict the cut-off values with the best proportion between sensitivity and specificity. Scatter plots were used to visualize ROC analyses with respective cut-off values. Cut-off values and scatter plots were only computed for PAS where ROC resulted in an area under the curve (AUC) significantly different from 0.5 (which represents chance).

Retrospective analyses (Spearman correlation, alpha = 0.05) showed age to be associated with PAS 3, Hn, and LZV. Therefore, authors performed partial correlations correcting for age for those three variables.

Statistical analyses were performed using SPSS 26 (SPSS Inc., Chicago, IL, USA) and pairwise deletion was used for missing items. Alpha was set at 0.05, and the Holm method [37] was applied for multiple comparisons. The method was applied separately for comparisons of the GMFM-66 with PAS (7 comparisons) and with pattern complexity (4 comparisons), as these were treated and interpreted as two different constructs.

## 3. Results

A convenience sample of 25 patients was included, and there were no missing items. The Kolmogorov–Smirnov test showed that PAS 5, PAS 6, PAS 4–6, and SampEn were not normally distributed. Visual inspection revealed that other variables probably differed from normal distribution. For reasons of consistency and better readability, all results were reported as median and interquartile range (IQR), and non-parametric testing was used.

The median age was 14.1 (IQR 10.4–16.6) years. Patient characteristics are shown in Table 2. GMFM-66 scores ranged from 38.7 to 100; the median score was 78.3 (IQR 63.3–88.0).

A mean of 9.4 (SD 0.6) hours was monitored with the IMUs. Example barcodes for children with different capacity levels are shown in Figure 1. Table 3 shows that a median of over 68% of the measurement time was spent in a sitting or lying position (PAS 1), 22% standing (PAS 2), and 8% active (PAS 3–6). All participants spent some of their time in an active state of short duration (PAS 3). Five participants did not achieve an active state of moderate duration (PAS 4), an additional nine participants (total 14) did not achieve an active state of long duration (PAS 5). Only three participants achieved an active state of very long duration (PAS 6).

Sample entropy, Lempel-Ziv complexity, standing (PAS 2), and active states of short and medium duration (PAS 3 and PAS 4) were not associated with gross motor capacity (GMFM-66). Comparison of GMFM-66 score with composite PA complexity resulted in a fair correlation, and the information entropy (Hn), the active state of long duration (PAS 5), as well as the grouping variables of the PAS in a moderate correlation (see Table 3). Lying/sitting (PAS 1) showed a tendency to be negatively associated with GMFM-66. A scatterplot matrix of the correlation is shown in Figure S1.

Age had a fair negative correlation with activities of short duration (PAS 3, rho = −0.398, *p* = 0.049) and a moderate negative correlation with SampEn and LZC (rho = −0.553, *p* = 0.004; rho = −0.514, *p* = 0.009). When controlling for age, correlations of GMFM−66 with PAS 3 and SampEn changed minimally and remained non-significant (r = 0.309, *p* = 0.142; r = 0.342, *p* = 0.102), indicating that age had a negligible influence on these relationships. However, partial correlation controlling for age of GMFM-66 with LZC resulted in a significant and moderate positive correlation (r = 0.531, *p* = 0.008).

**Figure 2:**
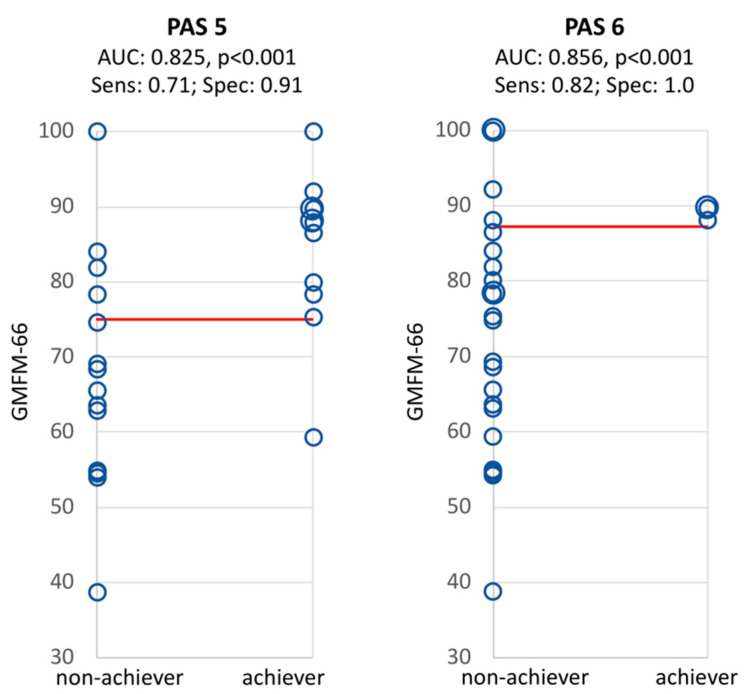
Receiver operating characteristics of PAS 5 and PAS 6. Sensitivity is defined as the proportion of positives (i.e., non-achiever) correctly identified as such. Specificity is defined as the proportion of negatives (i.e., achiever) correctly identified as such. The red lines indicate the cut-off levels for GMFM-66 scores with the best proportion of sensitivity and specificity to discriminate between achiever and non-achiever. Abbreviations: PAS, physical activity state; AUC, area under the curve; Sens, sensitivity; Spec, specificity; GMFM, Gross Motor Function Measure.

ROC analysis was not significant for PAS 4. Scatter diagrams for the ROC analyses of PAS 5 and 6 (both significant) are shown in Figure 2. The cut-off level to discriminate between achiever and non-achiever was a GMFM-66 score of 75.0 for PAS 5 and 87.3 for PAS 6.

## 4. Discussion

Main results showed that children spent 90% of their time sitting or standing and most of them did not achieve any active phase exceeding three minutes. When comparing performance with the GMFM-66, a measure of gross motor capacity, we found mostly fair to moderate correlations between participants’ capacity and daily life performance. However, ROC analyses revealed that a certain capacity level (cut-off level computed using the highest Youden Index) seems to be a prerequisite to enable PA of longer duration.

### 4.1. General Gross Motor Capacity and PA Level

GMFM-66 scores per GMFCS level found in this study (Table 2) were comparable with the GMFM-66 limits of the development curves found by Rosenbaum et al. [38] (87.7 for GMFCS I, 68.4 for GMFCS II, 54.3 for GMFCS III) indicating a representative sample for the three GMFCS levels regarding gross motor function.

The example PA barcodes in Figure 1 show the variability in intensity (different barcode colors) and complexity (amount of change between different barcode states) of PA in children with CP. In general, higher intensity PA as well as complexity augments with higher motor capacity. However, patients with similar motor capacity (e.g., examples 02, 03, 05, and 08) can have similar GMFM scores but very different daily life PA behavior. The same figure illustrates how valuable complexity metrics are; while examples 04 and 05 spent a similar proportion of their day active, their PA pattern complexity differs considerably, resulting in almost double CDS for example 05 compared to example 04. Similarly, the CDS of participants with similar CC (examples 01, 03, and 06) diverges when they show different proportions of sedentary and active behavior (% active). Finally, patients can have similar CDS (examples 02 and 04), despite their dissimilar CC, if they have inverted proportions of active behavior (i.e., the patient with higher CC, has a lower value of % active). Thus, complexity metrics and global activity metrics (lying/sitting, standing, active, etc.) are complementary and both valuable for clinicians to understand a patient’s PA performance, while the CDS can serve as an overall PA behavior score, which is especially interesting for research purpose (e.g., understanding PA behavior across various community dwelling/clinical populations, evaluation of treatment effects).

PAS computation disclosed that sedentary time of children in our study (68.2%, Table 3) was higher when compared to findings in Capio et al. [26] (around 55%, computed from hip-fixed Actigraph), comparable with the recent findings of Halma et al. [39] (72.1%, computed from Actigraph) and Bar-Haim et al. [24] (75.8%, thigh-fixed Activ-PAL), and lower compared to the values of Verschuren et al. [7] (76–99%, from a review including mostly adolescents and adults with CP). In general, comparisons between our novel approach compared to those reported in earlier studies are difficult because of differences in study populations, thresholds, and equipment, as well as PA computation. Especially comparison of the standing and active PAS with previous literature was limited, since our method had never been used in children with CP.

However, with two different approaches to separate our PAS in light and moderate to vigorous PA, our results are comparable to the ones found by Verschuren et al. [7]; in approach one, we count standing and PAS 3 as light and PAS 4–6 as moderate to vigorous PA, and in approach two, we count only standing as light, while all “active” PAS (i.e., PAS 3–6) as moderate to vigorous PA. Results of approach one correspond to the lower bound (2%), and results of approach two are similar to the higher bound (7%) of moderate to vigorous PA found by Verschuren et al. [7] and to the percentage of moderate to vigorous PA found by Capio et al. [26].

### 4.2. Relationship between Gross Motor Capacity and PA States and Pattern Complexity

Not all performance variables were correlated with the GMFM-66 score.

In general, higher capacity was associated with higher PA pattern complexity but the composite complexity (CDS) resulted only in fair and sample entropy in no correlation with the GMFM-66. Contrary to our hypothesis, lower capacity did only show a tendency for fair association with more sedentary behavior after the Holm method for adjusting *p*-values, while higher capacity was associated with more time spent in an active state of long duration and in the combined PAS (PAS 3–6 and PAS 4–6).

As the PA pattern complexity analysis is a novel approach that has not been used previously in children with CP, we cannot compare our findings to existing literature. Values of participants with CP in our study are comparable with values of elderly people with severe pain included in the study by Paraschiv-Ionescu et al. [28]. Patients with higher capacity, seem to have more complex PA patterns. However, strength of correlations and scatter plot interpretation (Figure S1) led us to assume that other factors such as intrinsic motivation to perform PA, fear of falling, or family habits in daily life have a substantial influence on PA performance. Therefore, complexity metrics might add valuable information when looking at a person’s daily life performance.

As we had hypothesized, we found some relationship but no systematic associations between motor capacity and global metrics of PA. Only two studies used a sufficiently similar methodology to allow a comparison with our results.

Bania et al. [23] found that more time spent in a sitting and lying position was associated with lower capacity, measured with two dimensions of the GMFM-66 (standing dimension and walking, running, jumping dimension). Although not significant, we found the same direction of association.

Our findings of positive association between capacity of % total time walking/running are in line with the positive correlation of % walking with the GMFM-66 dimension walking, running, jumping found by Wittry et al. [25] comparing various StepWatch variables to clinic-based measures.

However, Wittry et al. and Bania et al. used GMFM dimension E and GMFM dimensions D and E, respectively, while we used the whole GMFM-66. In addition, while they used a single-sensor setup, we used several synchronized sensors for comprehensive information allowing the computation of PAS and visualization of the daily activity by a PA color barcode. Finally, measurement duration was distinctly shorter in our study (single school day vs. several measurement days).

### 4.3. GMFM-66 Cut-Off Level for Achievers and Non-Achievers of PAS 5 and 6

The high and significant AUC suggest that the GMFM-66 can predict with good sensitivity and specificity whether or not a child with CP might achieve a high PA performance level (PAS 5 or 6). The AUC was not significant for PAS 4, meaning that the GMFM is not a good classifier to separate achiever from non-achiever for this PAS.

However, it has been shown that ROC analyses must be interpreted with caution, especially for a small sample size [40]. Furthermore, there were only three achievers (all with GMFCS level I) for PAS 6, and our classification was trained with all data. Therefore, to strengthen the current results and allow for conclusive interpretation, this model will need to be validated with a larger sample in a future study. However, some interpretations and hypotheses can be developed.

Cut-off points in Figure 2 show the motor capacity required to achieve PAS of long (PAS 5) and very long duration (PAS 6). While, due to the small sample size, the cut-off points might not be final, results imply that it is rather unlikely for children to achieve PAS 5 if they have a GMFM-66 score lower than 75.0 and PAS 6 if they have a GMFM-66 score below 87.3. Furthermore, it seems that some children, even though theoretically capable of achieving an active state of long and very long duration (PAS 5 and PAS 6) do not spontaneously use this capacity in daily life. This is in line with recent studies by Carcreff et al. [12,41] where children with CP usually over-performed in the clinical setting compared to habitual daily life with respect to gait characteristics.

Our cut-off points are based on the spontaneous behavior of study participants in their habitual daily life situations. The fact that one child with rather low GMFM-66 score was misclassified as non-achiever for PAS 5 suggests that cut-off points for predicting motor capability, thus what a child is able to do (in contrast to what he/she spontaneously does) in its habitual environment would be much lower. For future studies, it would be interesting to look at cut-off points predicting motor capability and, based on the results and promoting changes in lifestyle, try to push performance towards the limits of capability.

In general, a sufficient baseline motor capacity seems to be a prerequisite for good functioning in daily life. Nevertheless, there is no direct link between the two since a lower motor capacity does not systematically lead to a more sedentary behavior, and children with high motor capacity do not always perform well in daily life. Improvements in motor capacity could allow for but might not automatically generalize to higher motor performance. Very recent data by Halma et al. [39], showing no correlation between changes in motor capacity and changes in motor performance after an intensive intervention, support this theory.

### 4.4. Clinical Considerations and Study Limitations

Our approach of looking at PA pattern complexity of the barcodes, as previously proposed for other populations [28,29], is completely new in children with CP. We believe that this approach outreaches simple activity count, as it takes into consideration other aspects such as variety and temporal dynamics of barcode states. However, it is difficult to draw definite conclusions, as there is no literature to compare with and further validation of such measures in the study population as well as in healthy controls will be required.

One clear limitation of the current study was the short measurement time that might not be representative for habitual PA [18]. However, in a previous study with a very similar population, we showed good reliability for a single-day measurement with our approach [22], and none of the participants underwent intensified training or surgery between the measurements. It has to be mentioned that in the cited reliability study, only % of walking, standing, and lying/sitting was investigated, while further studies would be needed to determine the reliability of barcoding PA and PA complexity metrics. Another drawback is the time lap (average of 104 days) between the two measurements (GMFM-66 and IMU monitoring). This was due to the design of the two original studies. Another critical point is the sample size. Even though we included 25 participants, only three achieved the PAS 6, which weakens the power of statistical analyses with this parameter.

So far, no software is available that would allow routine data analyses by non-trained healthcare providers in usual clinical settings for our five-sensor set-up. This limits our approach, for now, to research settings, and patient acceptability of the set-up has to be further investigated. A single-sensor approach for locomotion and cadence detection has recently been developed and validated for children with CP in a semi-standardized environment [42]. In a future study, this approach could be transferred into real-life with the same population, and compared to our five-sensor set-up for validation. Methods to use single-sensor data for PA barcoding have been described and used in well-functioning older adults [29]. Providing these metrics with a single-sensor measurement could offer a simplified alternative to the comprehensive five-sensor set-up we used.

As mentioned above, efforts should be made to enable comparison of the same or at least more similar metrics between clinical and everyday life settings when comparing capacity with performance. Otherwise, conclusions from such comparisons will remain limited. However, measurement tools and routines for data analyses are currently lacking, especially in the heterogeneous population of children with CP [43].

## 5. Conclusions

Barcoding PA seems a promising outcome, making results visible and understandable for different stakeholders, including children themselves. Furthermore, the computation of complexity metrics in children with CP is a new approach and adds valuable information about daily life performance in this population.

Our results point out the importance of objective measures of activity performance to complement standard clinical assessments, as we found no strong association between the two. However, for higher PAS, the GMFM discriminated well between children with CP who might or might not achieve these PAS in their natural environment.

## Figures and Tables

**Table 1:** Barcode and physical activity states definition.

Mode	Duration (Sec)	Intensity (Body Acceleration or Cadence)	Barcode States	PAS
LYING or SITTING	-	Low	1	1
	-	Moderate	2	
STANDING	-	Low	3	2
		Moderate	4	
		High	5	
		Very High	6	
ACTIVE Walking or Running	Short (<60)	Slow (<70)	7	3
		Moderate (70–100)	8	
		Fast (100–130)	9	
		Very fast (>130)	10	
	Medium (61–120)	Slow (<70)	11	4
		Moderate (70–100)	12	
		Fast (100–130)	13	
		Very fast (>130)	14	
	Long (121–360)	Slow (<70)	15	5
		Moderate (70–100)	16	
		Fast (100–130)	17	
		Very fast (>130)	18	
	Very long (>360)	Slow (<70)	19	6
		Moderate (70–100)	20	
		Fast (100–130)	21	
		Very fast (>130)	22	

For LYING or SITTING and STANDING body acceleration, and for ACTIVE Walking or Running cadence is used to determine intensity. Abbreviations: sec, seconds; PAS, physical activity states.

**Table 2:** Patient characteristics.

Age (y), median (IQR)	14.1 (10.4–16.6)
Sex, N girls in group (%)	15 (60)
Topography (N)	
Hemiplegia	11
Diplegia	10
Tetraplegia	4
Type of CP	
Predominantly spastic	21
Spastic, dystonic	3
Spastic, ataxic	1
GMFCS level (N)	
I	13
II	4
III	8
GMFM-66, median (IQR)	
GMFCS I	88.0 (81.0–90.9)
GMFCS II	75.0 (67.9–77.6)
GMFCS III	57.1 (54.2–63.5)
All	78.3 (63.3–88.0)

Abbreviations: y, years; IQR, interquartile range; CP, cerebral palsy; GMFCS, Gross Motor Classification System; N, number; GMFM, Gross Motor Function Measure.

**Table 3:** Physical activity pattern complexity and states and their correlations with gross motor capacity.

	GMFCS I			GMFCS II			GMFCS III			GMFCS All Levels			Correlation with	
	N = 13			N = 4			N = 8			N = 25			GMFCS, All Levels	
	Median	Q1	Q3	Median	Q1	Q3	Median	Q1	Q3	Median	Q1	Q3	Rho	*p*
**Pattern complexity**														
Hn	0.331	0.312	0.378	0.280	0.223	0.377	0.227	0.165	0.298	0.308	0.243	0.354	**0.568**	**0.003**
SampEn	0.010	0.006	0.022	0.010	0.007	0.023	0.007	0.003	0.013	0.009	0.005	0.019	0.241	0.246
LZC	0.091	0.076	0.105	0.088	0.078	0.123	0.062	0.054	0.083	0.083	0.073	0.103	0.368	0.071
CDS	14.12	11.42	24.91	9.22	5.43	20.10	6.05	2.83	14.84	13.25	7.05	18.74	**0.467**	**0.019**
**PAS**														
1—Lying or Sitting	66.8	56.7	71.3	75.1	62.1	83.4	80.2	62.3	87.7	68.2	59.0	78.3	−0.446 *	0.025
2—Standing	22.6	20.7	32.2	17.1	9.1	24.7	14.8	8.7	29.1	21.9	14.8	28.4	0.302	0.143
3—Active short	6.7	5.3	9.3	7.0	5.9	11.5	4.5	3.1	6.8	6.1	4.8	8.4	0.199	0.341
4—Active medium	1.3	0.6	1.9	0.6	0.0	1.3	0.2	0.0	1.1	1.0	0.2	1.5	0.364	0.074
5—Active long	0.7	0.0	3.2	0.2	0.0	1.8	0.0	0.0	0.0	0.0	0.0	1.0	**0.619**	**0.001**
6—Active very long	0.0	0.0	0.7	0.0	0.0	0.0	0.0	0.0	0.0	0.0	0.0	0.0	N.A.	N.A.
Active all (PAS 3–6)	11.1	7.4	12.2	8.7	6.6	13.2	5.1	3.3	7.5	7.9	6.2	11.4	**0.550**	**0.004**
PAS 4–6	2.5	1.3	6.5	0.9	0.0	3.1	0.2	0.0	1.1	1.3	0.2	3.1	**0.543**	**0.005**

Higher GMFM-66 scores are associated with higher Information Entropy (Hn), higher Lampel-Ziv complexity (LZC), higher composite complexity (CDS), and more time spent in activities of long duration (PAS 5) and, in general, in higher activity states (PAS 3–6, PAS 4–6). On the contrary, lower GMFM-66 scores tend to be associated with more time spent lying or sitting (PAS 1). PAS 6: Only three subjects achieved this PAS and for 22 subjects the value was 0. Therefore, no correlation was performed for this variable. Abbreviations: Hn, Information Entropy; SampEn, Sample Entropy; LZC, Lampel-Ziv complexity; CDS, composite deterministic score; PAS, physical activity states; IQR, interquartile range; rho, Spearman’s rho; N.A., not applicable. bold: significant using the Holm method, * tendency for correlation (*p* < 0.05).

## Supplementary Materials

The following are available online at https://www.mdpi.com/1424-8220/20/20/5861/s1, Supplementary document 1: Instructions for parents, Figure S1: Scatterplot matrix of correlations, Figure S2: ROC curves of PAS 5 and PAS 6.
