# Supplementary material for: Multidimensional Measures of Physical Activity and Their Association with Gross Motor Capacity in Children and Adolescents with Cerebral Palsy
Source: Sensors (Basel). 2020 Oct 16;20(20):5861. doi: 10.3390/s20205861 (PMC7589543; doi:10.3390/s20205861)

Liste du matériel fourni

- ☐ 5 capteurs Physilog avec leur sticker de fixation (+4 stickers de rechange)
- ☐ 1 protection plastique transparente
- ☐ 1 concentrateur USB (chargeur)
- ☐ 1 câble d'alimentation pour concentrateur USB
- ☐ 5 câbles de branchement

Aide à la mise en place des capteurs

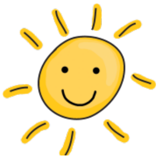

A faire le matin au réveil

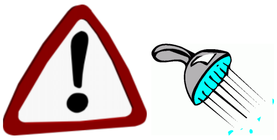

Les capteurs NE doivent PAS être portés sous la douche ou à la piscine

1) Placer les 5 capteurs sur une table

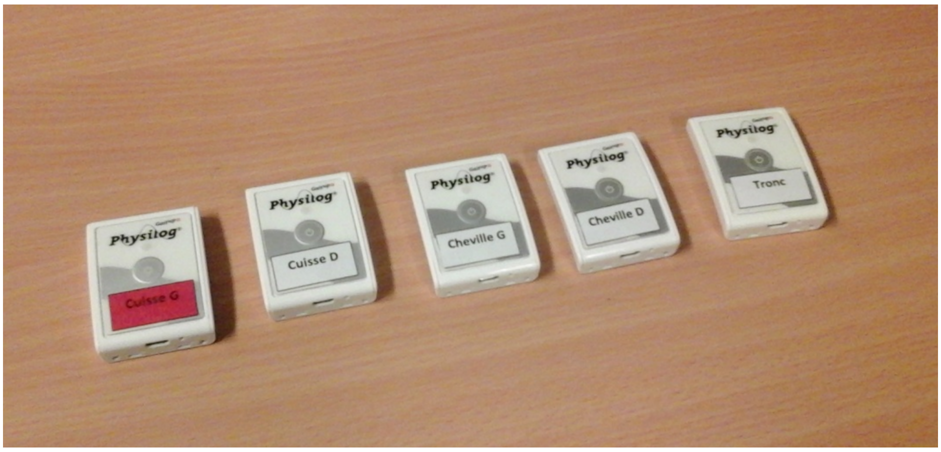

2) Allumer le capteur avec une étiquette rouge en premier puis allumer les 4 autres

Pour allumer un capteur :

- Appuyer sur le bouton 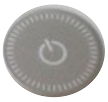 pendant 1 seconde:

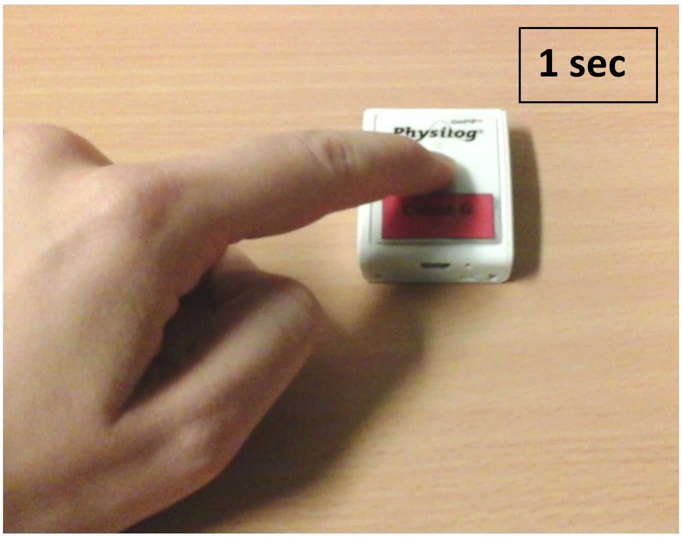

- Une lumière verte commence à clignoter :

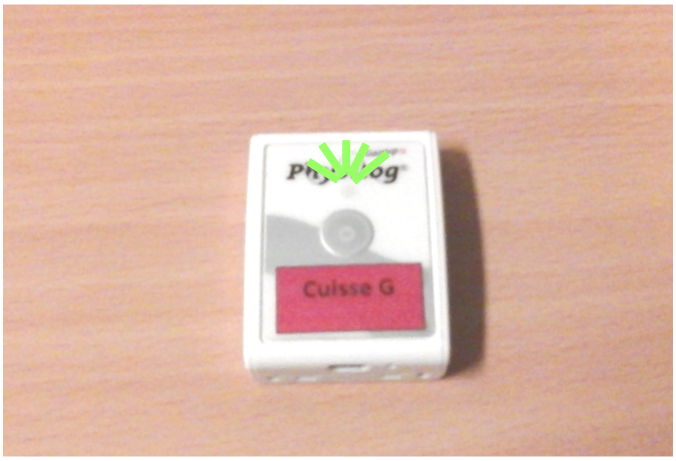

3) Une fois que tous les capteurs sont allumés, vérifier que les 5 clignotent vert **EN MÊME TEMPS** (de manière synchrone):

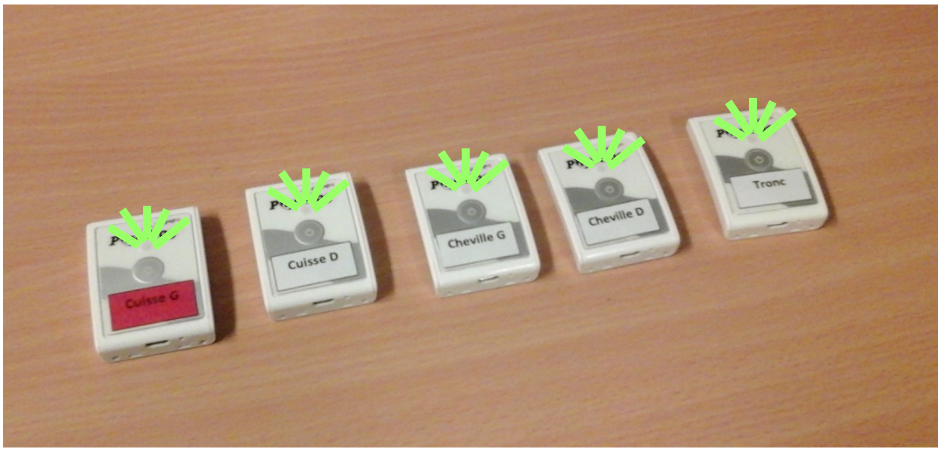

➔ S'ils ne clignotent pas en même temps. Eteindre tous les capteurs (voir 6, page 11) et recommencer.

4) Coller le capteur du tronc au milieu de la poitrine

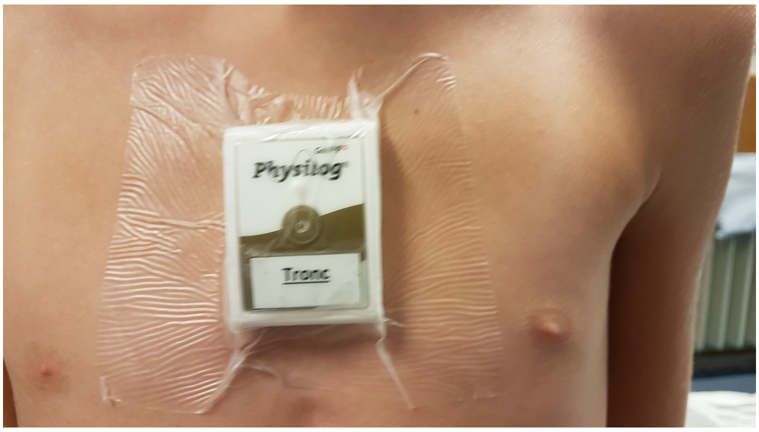

5) Placer les capteurs sur les jambes comme suit:

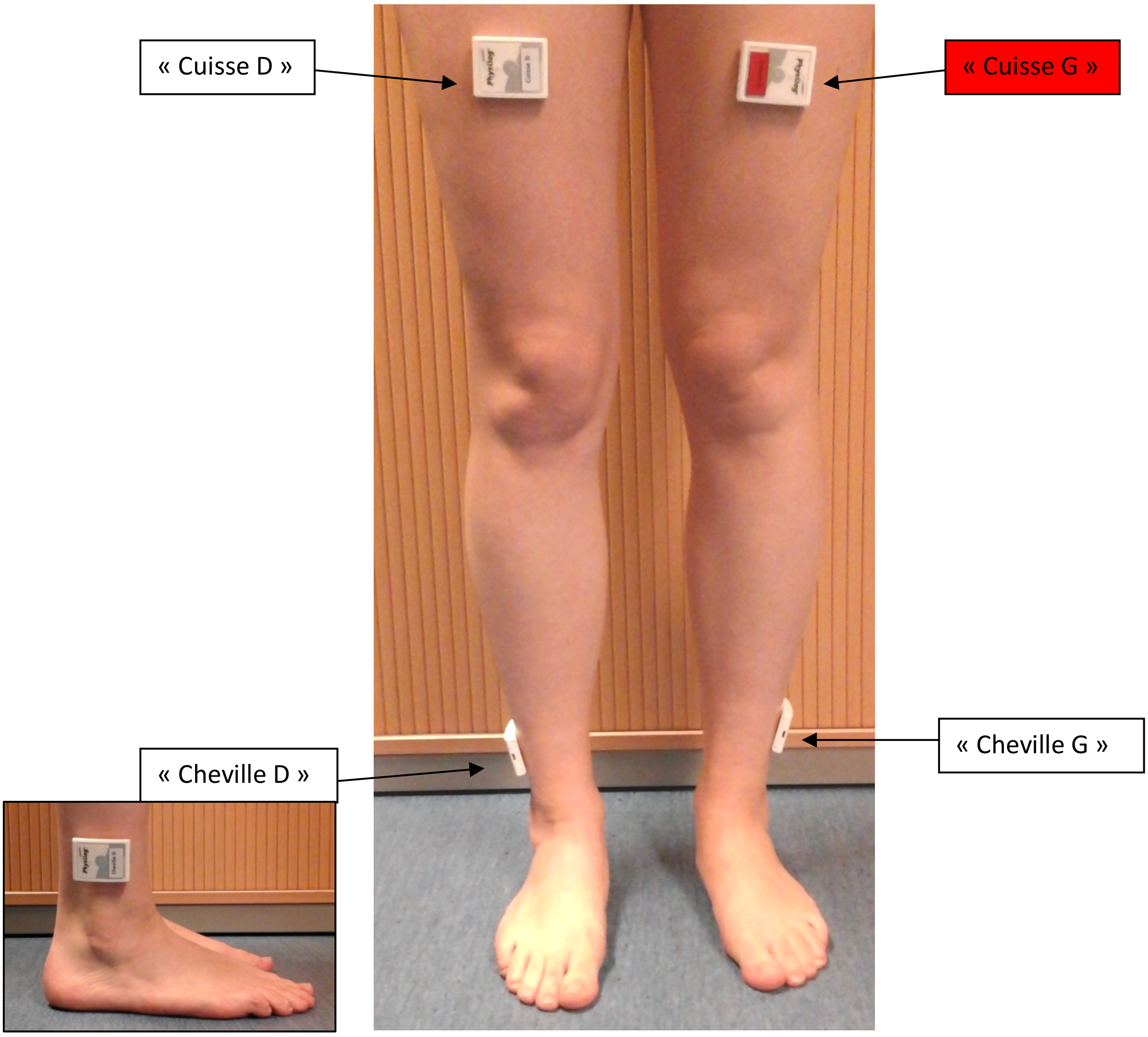

Astuce : Bien appuyer sur le capteur pour chasser l’air entre le capteur et la peau

Avec des orthèses, placer les capteurs comme suit:

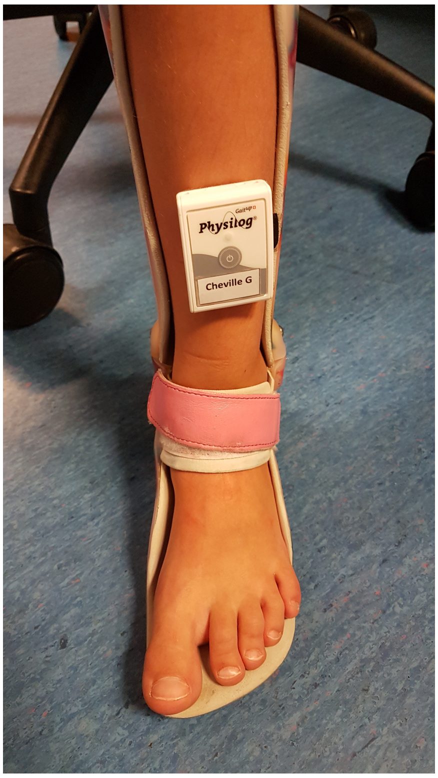

6) Une fois les capteurs fixés, rester couché bien aligné (si possible sur un tapis, pas sur un lit) pendant 30 secondes. Et puis rester debout le plus droit possible sans bouger pendant 30 secondes.

30 sec

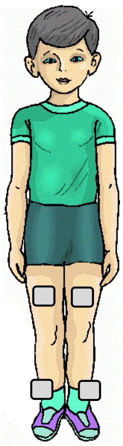

Et si possible, prendre une photo (à nous envoyer par la suite par email : corinna.gerber@chuv.ch)

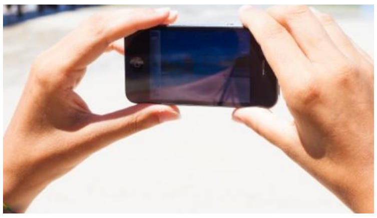

Astuce : Essayer de porter un short moulant sous le pantalon

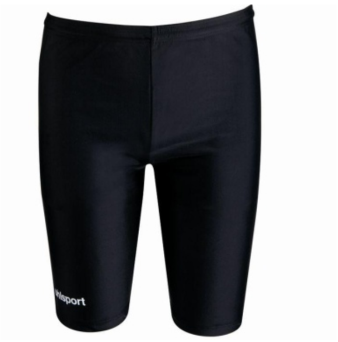

ou opter pour un pantalon bien serré au niveau des cuisses

Aide pour retirer les capteurs

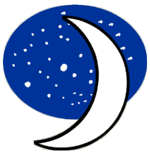

A faire le soir avant de se coucher

1) Avant de retirer les capteurs : rester debout le plus droit possible sans bouger pendant 30 secondes

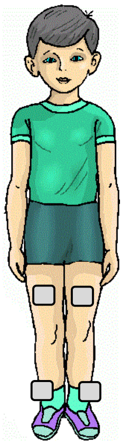

30 sec

2) Retirer délicatement les capteurs
3) Placer tous les capteurs sur une table

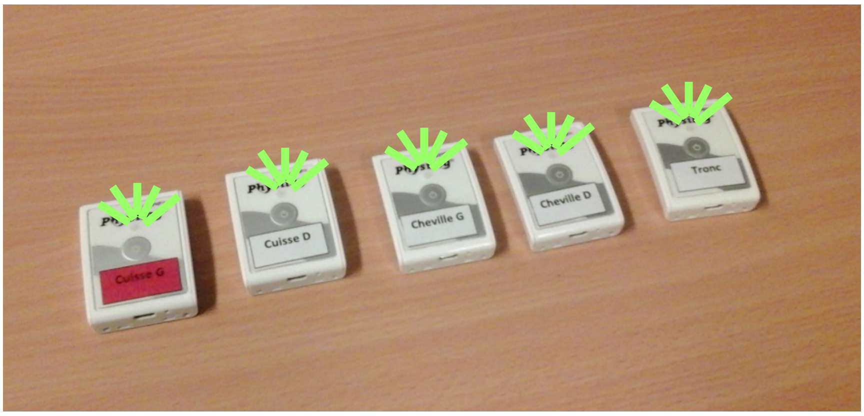

➔ Vérifier qu'ils clignotent toujours en même temps (sinon, noter sur le journal de bord quel capteur semble avoir un problème)

4) Eteindre les capteurs avec étiquette blanche en premier (ordre sans importance) puis celui avec une etiquette rouge

Pour éteindre : appuyer sur le bouton 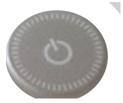 jusqu'à ce qu'une lumière rouge apparaissent sur le capteur.

5) Mettre les capteurs à recharger pendant la nuit :

- Brancher le concentrateur USB à une prise secteur
- Brancher chaque capteur au concentrateur USB par l'intermédiaire des câbles gris
- Vérifier qu'ils clignotent tous rouge (pas forcément en même temps). Lorsque le capteur clignote vert c'est qu'il est complètement rechargé.

Aide à l’utilisation des capteurs

Pour tous problèmes, ne pas hésiter à contacter Corinna au (+41)21 314 41 06

Corinna.gerber@chuv.ch

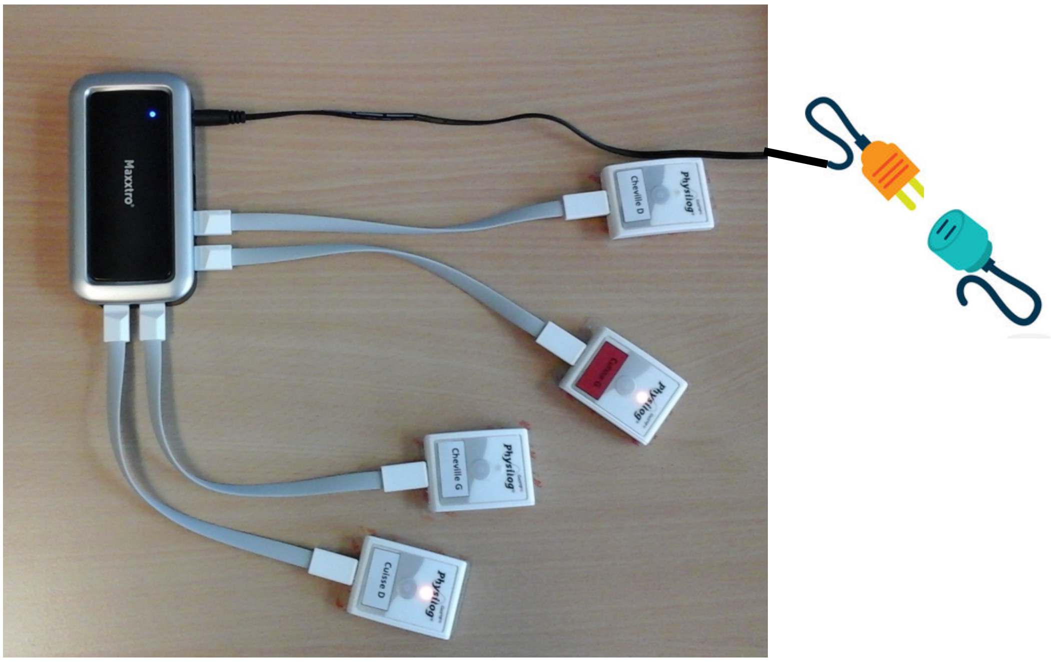

Supplement: Supplementary file 1 [file sensors-20-05861-s001.zip › Supplementary document 1.pdf]
