# Supplementary figures and images for: Multidimensional Measures of Physical Activity and Their Association with Gross Motor Capacity in Children and Adolescents with Cerebral Palsy
Source: Sensors (Basel). 2020 Oct 16;20(20):5861. doi: 10.3390/s20205861 (PMC7589543; doi:10.3390/s20205861)

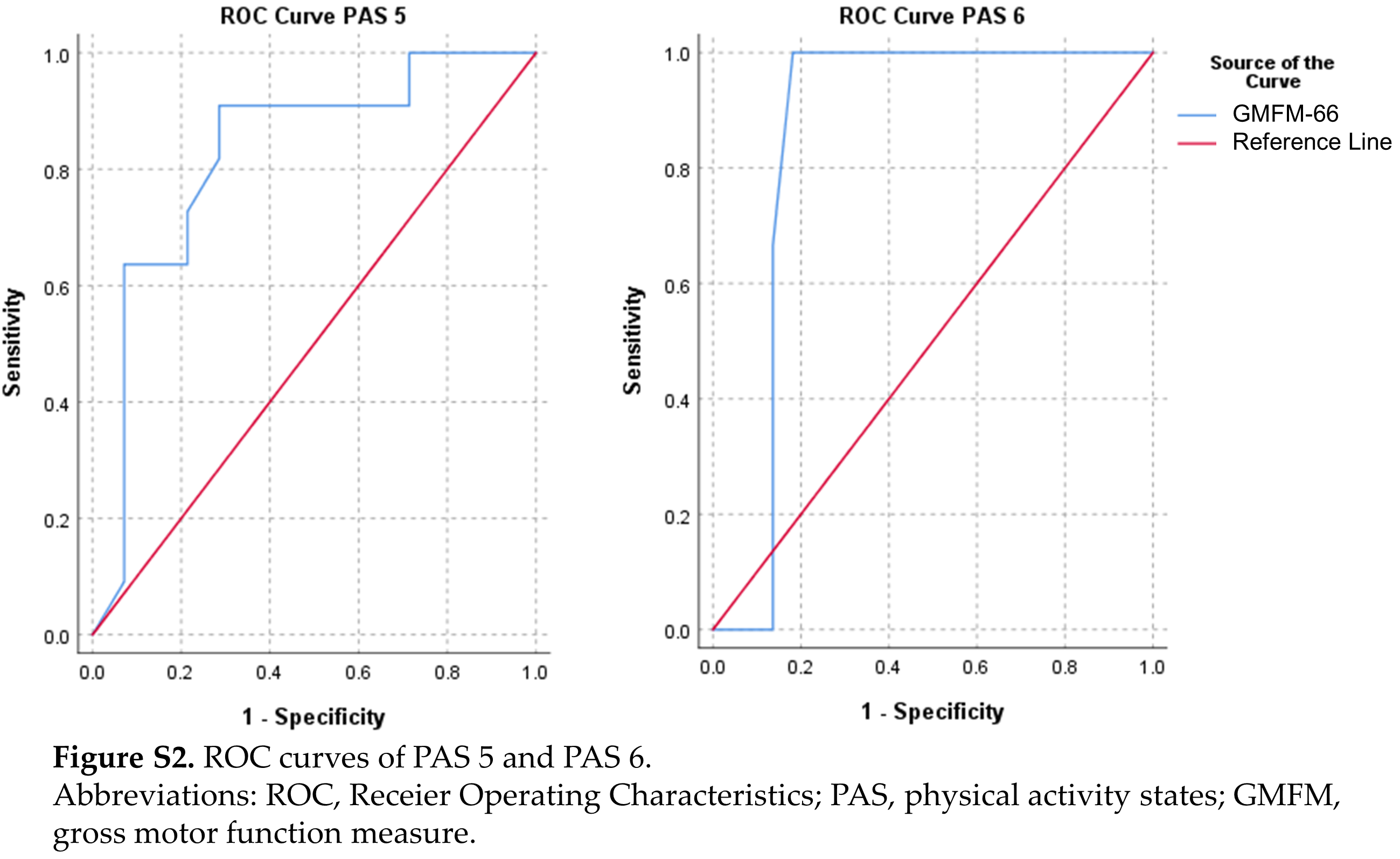

Supplement: Supplementary file 1 [file sensors-20-05861-s001.zip › Figure S2.PNG]

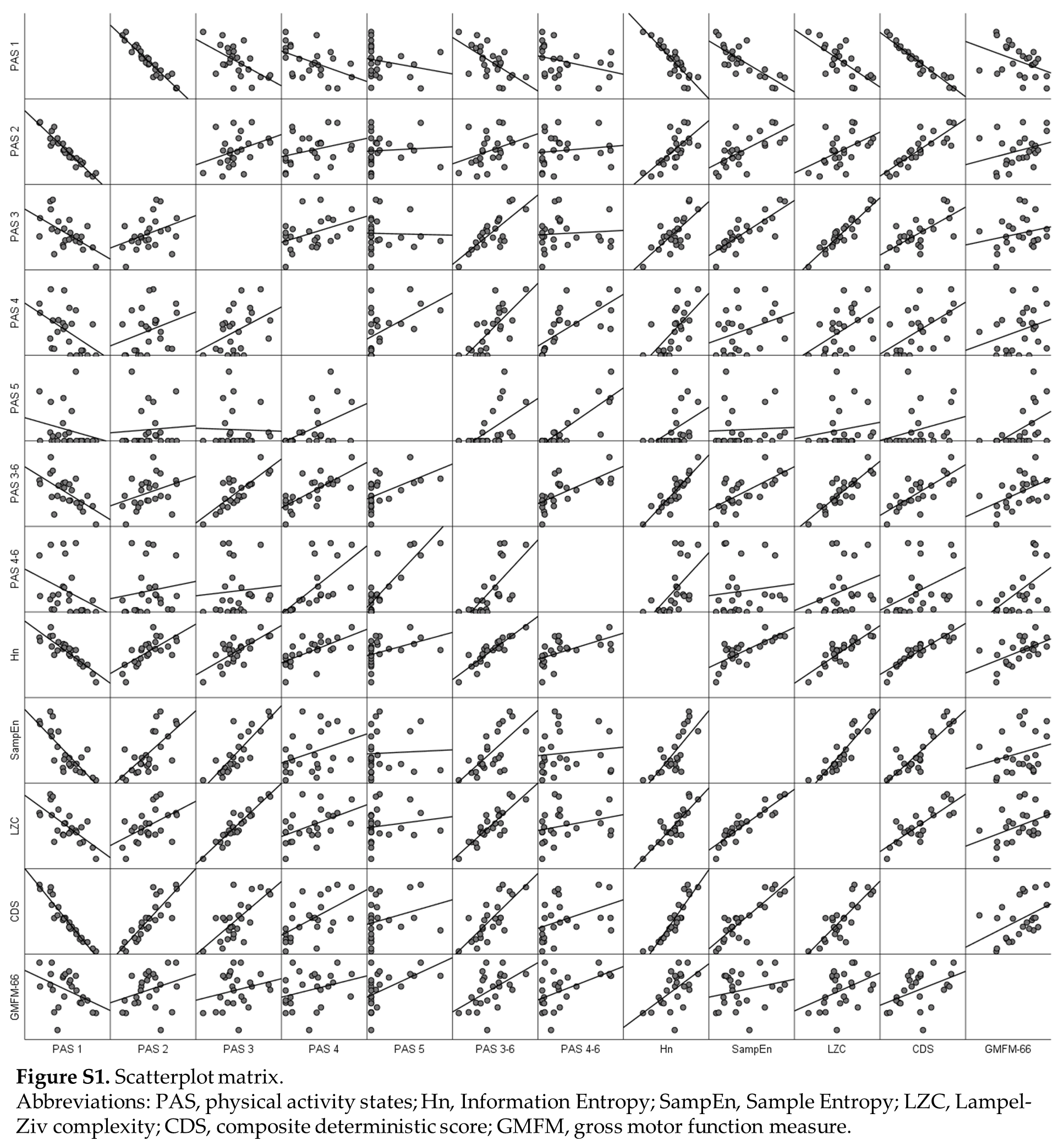

Supplement: Supplementary file 1 [file sensors-20-05861-s001.zip › Figure S1.PNG]
